# Nutrient adequacy during weight loss interventions: a randomized study in women comparing the dietary intake in a meal replacement group with a traditional food group

**DOI:** 10.1186/1475-2891-6-12

**Published:** 2007-06-25

**Authors:** Judith M Ashley, Holly Herzog, Sharon Clodfelter, Vicki Bovee, Jon Schrage, Chris Pritsos

**Affiliations:** 1 Nutrition Department, University of Nevada, Reno, Nevada 89557, USA; 2 Washoe County District Health Department, Reno, Nevada 89520, USA; 3 Western Bariatric Institute, Reno, Nevada 80503, USA; 4 Kansas University Medical Center, Wichita, Kansas 67214, USA

## Abstract

**Background:**

Safe and effective weight control strategies are needed to stem the current obesity epidemic. The objective of this one-year study was to document and compare the macronutrient and micronutrient levels in the foods chosen by women following two different weight reduction interventions.

**Methods:**

Ninety-six generally healthy overweight or obese women (ages 25–50 years; BMI 25–35 kg/m^2^) were randomized into a Traditional Food group (TFG) or a Meal Replacement Group (MRG) incorporating 1–2 meal replacement drinks or bars per day. Both groups had an energy-restricted goal of 5400 kJ/day. Dietary intake data was obtained using 3-Day Food records kept by the subjects at baseline, 6 months and one-year. For more uniform comparisons between groups, each diet intervention consisted of 18 small group sessions led by the same Registered Dietitian.

**Results:**

Weight loss for the 73% (n = 70) completing this one-year study was not significantly different between the groups, but was significantly different (p ≤ .05) within each group with a mean (± standard deviation) weight loss of -6.1 ± 6.7 kg (TFG, n = 35) vs -5.0 ± 4.9 kg (MRG, n = 35). Both groups had macronutrient (Carbohydrate:Protein:Fat) ratios that were within the ranges recommended (50:19:31, TFG vs 55:16:29, MRG). Their reported reduced energy intake was similar (5729 ± 1424 kJ, TFG vs 5993 ± 2016 kJ, MRG). There was an improved dietary intake pattern in both groups as indicated by decreased intake of saturated fat (≤ 10%), cholesterol (<200 mg/day), and sodium (< 2400 mg/day), with increased total servings/day of fruits and vegetables (4.0 ± 2.2, TFG vs 4.6 ± 3.2, MRG). However, the TFG had a significantly lower dietary intake of several vitamins and minerals compared to the MRG and was at greater risk for inadequate intake.

**Conclusion:**

In this one-year university-based intervention, both dietitian-led groups successfully lost weight while improving overall dietary adequacy. The group incorporating fortified meal replacements tended to have a more adequate essential nutrient intake compared to the group following a more traditional food group diet. This study supports the need to incorporate fortified foods and/or dietary supplements while following an energy-restricted diet for weight loss.

## Background

With the current incidence of both overweight and obesity in adults in the United States at 65%, health care practitioners need to play an even greater role in weight control [1]. Management involves not only weight loss and maintenance of body weight, but also measures to control other health risk factors. Patient financial considerations also play a part, including the cost of travel to the clinical facility, time lost from work, and any weight counseling that is not covered by health insurance. Guidelines based on recent evidence suggest that small weight losses (5 to 10% of initial body weight) can improve obesity-related health complications [2].

Since a major health goal is to achieve and maintain a desirable weight, there is a heightened awareness of the types of diets used to achieve weight control [3]. Incorporating meal replacements (1–2 per day) into traditional lifestyle interventions has been shown to be effective in treating overweight or obese patients, and this option is currently recommended for health practitioners [4,5]. Although efficacy is one important criteria in evaluating weight control studies, an equally important concern is safety, including the adequacy of the dietary intake as it impacts on long-term health [6]. This study is a follow-up to our published research showing the effectiveness of meal replacements in weight loss and in improving clinical health risks. In the second year follow up of this previous research, we found some positive trends in the nutrient adequacy for women who incorporated meal replacements as a weight loss strategy [7,8]. However, dietary change was not a primary measure in our previous research, which was designed to examine the correlations of weight loss with clinical risk factor changes.

This current study was undertaken to extend the preliminary results on alterations in diet in women using meal replacements as a weight loss intervention strategy. The objective of this one-year study was to document and analyze the nutritional adequacy of a traditional food-group diet intervention compared to a traditional food-group diet intervention that also incorporated meal replacements as a strategy for weight loss.

## Methods

### Participants

Ninety-six generally healthy overweight/obese women, ages 25 to 50 years were selected from those responding to media recruitments in the local university community. Women were eligible if they were overweight to mildly obese as defined by a BMI (kg/m^2^) between 25 and 35 [6]. Potential candidates attended a group orientation meeting where the study was explained to them before written consent was obtained. Exclusion criteria included current chronic or psychological disease, abnormal serum laboratory values of clinical significance, or current hormone-replacement therapies. Women were also excluded who were pregnant, planning to become pregnant, lactating, planning to move out of the area within the following year, or currently participating in a weight loss program. Eligible women had an initial (baseline) one-hour clinic assessment to determine their fasting blood chemistry values (to rule out disease risk), body measurements (weight, height, BMI, waist circumference, body fat percentage), dieting and physical activity habits, and psychosocial status. There were no costs to participants for study assessments and interventions. The University of Nevada Human Subjects Protection Committee (Institutional Review Board) approved all procedures used in this study. All subjects completed this university-based intervention during the years 2002 to 2004.

### Lifestyle Dietary Intervention

Subjects were assigned to 1 of 2 interventions: a Meal Replacement Group (MRG) or a Traditional Food Group (TFG). Subjects in both groups attended the same total number of classes (eighteen), including bimonthly classes (twelve) for the first six months, followed by monthly classes (six) for the last six months of intervention. Subjects received instruction materials developed by the study's Registered Dietitian (RD). They also received the LEARN Program manual for Weight Control, with the MRG participants receiving the separate LEARN Program Meal Replacement Edition manual [9]. For a more uniform comparison between groups, the same RD taught all sessions for both groups. Diet instruction for all subjects included a low energy diet of approximately 5040 kJ/day (1200 kcal/day).

### Group 1: Traditional Food Group (TFG)

Subjects in the TFG were instructed in a diet plan based on self-selection of conventional foods for meals and snacks using the USDA Food Guide Pyramid. They were encouraged to include the recommended servings of all food groups, including fruits and vegetables with meals and snacks. To enhance compliance and to equalize the monetary value of providing free meal replacements in the Meal Replacement Group (MRG), the women in this group (TFG) received free grocery store gift certificates ($20/month) at their intervention group meetings. This monetary incentive enabled them to redeem the gift certificates for their choice of "healthy" foods at their local food stores.

### Group 2: Meal Replacement Group (MRG)

Subjects in the MRG were instructed in a diet plan consisting of commercially available meal replacements, as well as a self-selection of conventional foods using the USDA Food Guide Pyramid. Two of the three main meals (breakfast, lunch, dinner) were replaced with study meal replacement drinks or meal replacement bars (Unilever; Slim-Fast Nutrition Institute, USA). Because this study lasted for one year, subjects were given a choice of the study brand of meal replacement drinks or meal replacement bars for a greater variety to help with compliance over the one year intervention period. At their intervention group meetings, the women received a supply of free vouchers that they could easily carry and redeem for their choice of the study brand of meal-replacement drinks and/or bars that were readily available at their local food stores. By having a supply of these free vouchers, the women were able to conveniently pick up their study brand of products when they did their normal food shopping during the week.

Each study liquid meal replacement drink contained 924 kJ (220 kcal), 7 to 10 grams of protein, 40 to 46 grams of carbohydrate, 5 grams of dietary fiber, and 1.5 to 3 grams of fat. Subjects were able to choose from the study brand of either milk-based and soy-based meal replacement drinks in a variety of flavors. The study brand of fortified meal replacement drinks contained 15–100% of the % Daily Value (%DV) for essential vitamins and minerals. Subjects could also choose from the study brand of a variety of meal replacement bars. The study brand of meal replacement bars contained approximately the same 924 kJ (220 kcal) as the drinks, with 8 grams of protein, 33 to 36 grams of carbohydrate, 2 grams of dietary fiber, and 5 grams of fat. These fortified study brand of meal replacement bars contained 25%–35% of the % Daily Value (%DV) for essential vitamins and minerals. In addition to the two study brand meal-replacements, it was recommended that the women incorporate the dietary food guide levels of fruits and vegetables as snacks, along with one moderately low fat, low energy meal each day.

### Dependent Outcome Measures

Assessment outcome measures were taken at baseline, 6 months and 12 months and included weight, height, waist circumference, and body composition. Fasting blood was taken by a certified phlebotomist to measure blood chemistries. Prior to randomization, the study physician reviewed the subject's blood chemistries to screen out any subjects that might have an abnormal blood laboratory value of clinical significance that might potentially exclude her from the study. No subjects were excluded based on the physician's evaluation of their baseline blood chemistries. Weight measurements and body fat measurements were taken using a computerized bioelectrical impedance scale (Tanita, San Diego, CA). Height was measured using a mounted wall stadiometer, and BMI (kg/m^2^) was calculated. Waist circumference was measured at the narrowest point of the torso using a non-stretchable measuring tape. Blood pressure was measured while the subject was seated using a digital manometer machine. Three blood pressure measurements were taken, with the average of the last two values recorded. Subjects kept a food diary of their self-selected foods and beverages for three consecutive days (one weekend day and two weekdays) prior to each assessment day. Nutrient analysis on the three-day food records was done using a licensed copy of the Minnesota Nutrient Data System Research program (NDS, 2001, version 4.03). The number of daily servings of fruits and vegetables and meal replacements were calculated directly from the subject's three-day food records.

### Statistical Analysis

The study design was initially based on a sample size of 96 subjects which would yield a power of 80% assuming a two-sided type I error rate of 5%. This accounted for a standard 20% attrition rate. Although the study final attrition rate was 27% at one year, statistically significant differences in nutrient intake variables were found between and within groups comparing baseline with 6 months and one year. However, this does not rule out that some statistically significant differences existed that were not found in the final data analysis results. The primary outcome was weight lost over a 1-year period. The analyses on all enrolled subjects (n = 96) were conducted using two intent-to-treat methods: 1) last weight carried forward, and 2) baseline weight carried forward. Both methods indicated similar results. The remaining analyses were conducted on only those subjects who completed the two follow-up assessment visits (n = 70). All analyses comparing the between group differences used an independent t-test. Those analyses focused on change within each group used a paired t-test. If a baseline between group significance was found, an adjustment for baseline values using regression analysis assessed whether the significant findings were still valid. All findings identified as significant were supported by this analysis. All analyses conducted utilized SPSS software (version 11.0, Chicago, IL). Variable results are given as mean ± standard deviation (sd), with significance determined at p ≤ 0.05. A separate analysis of the group prevalence of inadequacy using the EAR cut-point method was done using the SIDE software program from Iowa State University [10].

## Results

### Subjects completing the one-year study

Of the 96 initial female subjects who were randomly assigned to study groups, 70 subjects (or 73 percent) completed the one-year study, with equal retention in each group (n = 35). No adverse events were reported or associated with any women who dropped out of the study. Baseline characteristics of the subjects by groups are shown in Table 1. The two significant differences among the parameters by the treatment group (age, body fat) were assessed using regression analysis to determine that milestone significant findings at 6 and 12 months were still valid.

**Table 1:** Baseline characteristics of both intervention groups

***Variable***	**TFG (n = 35)**	**MRG (n = 35)**
Age (years)	39.79 (6.1)*	36.7 (6.3)*
Weight (kilograms, kg)	78.9 (10.6)	79.2 (7.4)
BMI (kg/m^2^)	29.5 (3.1)	29.1 (2.4)
Body Fat (%)	47.9 (7.9)*	44.6 (5.8)*
Waist Circumference (cm)	90.1 (6.6)	89.7 (7.2)
REE (calculated)	1483.6 (126.5)	1505.8 (78.7)

* Significant differences (p < 0.05) between groups at baseline

Mean ± (standard deviation)

TFG = Traditional Food Group; MRG = Meal Replacement Group

Group variable changes are shown in Table 2. Although mean weight loss was not significantly different between the two groups, further analysis showed a significant difference at one-year within each group. There was a significant mean weight loss in the TFG of -6.1 ± 6.7 kg (-13.5 ± 14.7 pounds) or -8.0% ± 8.4% compared to baseline weight. The MRG showed a significant mean weight loss of -5.0 ± 4.9 kg (-11.0 ± 10.8 pounds) or -6.2% ± 6.3% compared to baseline weight. Although not a significant change, there was a decrease in percent body fat and waist circumference in both groups.

**Table 2:** Changes in both group variables from baseline

***Variable***	**TFG (n = 35)**	**TFG (n = 35)**	**MRG (n = 35)**	**MRG (n = 35)**
	6 mo – baseline	Yr1 – baseline	6 mo – baseline	Yr1 – baseline

Weight (kilograms, kg)	-5.4 (5.4)	-6.1 (6.7)	-5.2 (4.0)	-5.0 (4.9)
BMI (kg/m^2^)	-2.1 (2.2)	-2.6 (2.9)	-1.9 (1.4)	-1.9 (1.9)
Body Fat (%)	-5.4 (6.5)	-5.7 (8.1)	-4.3 (4.0)	-4.5 (6.5)
Waist Circumference (cm)	-6.1 (5.5)	-6.1 (7.0)	-4.8 (3.22)	-4.9 (4.6)
REE (calculated)	-52.2 (51.1)	-75.6 (83.0)	-49.6 (36.4)	-50.2 (47.1)

* Significant difference (p ≤ 0.05) between groups from baseline

REE = Resting Energy Expenditure

Mean ± (standard deviation)

TFG = Traditional Food Group, MRG = Meal Replacement Group

### All subjects enrolled in the study (intent-to-treat)

Year-end changes in weight were also examined in the intent-to-treat model. This analysis included all subjects (n = 96) enrolled in the study, whether or not they completed the one-year intervention. The analysis was based on the available data for all randomized subjects (n = 96) with the last weight observation carried forward. No significant difference in weight loss between groups was found with this additional analysis. Therefore, similar conclusions from this intent-to-treat analysis were made, although the weight loss was not of the same magnitude as for the subjects who completed the one-year study.

### Nutrient mean intake changes from three-day food records

Mean energy, macronutrient and micronutrient changes are shown in Tables 3, 4, and 5 for the Traditional Food Group (TFG) and the Meal Replacement Group (MRG). For energy, the self-reported mean dietary intakes fell as expected below the DRI Acceptable Macronutrient Distribution Range of 10800 kJ/day (2400 kcal/day) [11]. However, neither group quite reached the targeted reduced energy intake goal of 5400 kJ/day (1200 kcal/day) in this weight loss intervention. For macronutrient percentages, the ratios (carbohydrate: protein: fat) were within the DRI recommendation ranges at the study time points. For saturated fat, the mean intake was slightly lowered to ≤10%, although still higher that the recommendation of ≤7%. For mean dietary fiber intake, there was an increase in intake from baseline for both groups, with a significant difference at 6 months, as well as a significant increase within the MRG at 6 months. However, both groups fell short of the recommended 25 grams/day.

**Table 3:** Changes in macronutrient and fiber intake in both groups from baseline

***Variable per day***	**TFG (n = 35)**	**TFG (n = 35)**	**TFG (n = 35)**	**MRG (n = 35)**	**MRG (n = 35)**	**MRG (n = 35)**
	**Baseline**	**6 month**	**Year 1**	**Baseline**	**6 month**	**Year 1**

Energy (kilojoules, kJ)	8047 (2356)	6124 (1583)	5729 (1424)	8408 (2213)	5838 (1621)	5993 (2016)
Energy (kilocalories, kcal)	1916 (561)	1458 (377)	1364 (339)	2002 (527)	1390 (386)	1427 (480)
Carbohydrates %	48.3 (8.2)	49.9 (8.3) ^b^	50.1 (10.6)	49.5 (7.6)	56.6 (8.7)^b^	55.04(11.6)
Protein %	15.1 (3.8)	19.2 (4.1)	19.2 (4.6) ^c^	15.4 (3.2)	17.8 (4.1)	16.8 (3.1) ^c^
Fat %	35.3 (6.6)	30.7 (6.9)	31.0 (8.1)	35.35 (6.7)	27.36 (7.4)	29.41 (9.8)
Saturated Fat %	11.9 (3.3)	10.4 (2.4)	10.2 (3.2)	12.0 (2.5)	9.5 (2.9)	9.8 (3.3)
Cholesterol (mg)	200 (104) ^a^	199 (122)	193 (91)	261 (117) ^a^	156 (103)	165 (126)
Dietary Fiber (g)	5 (2)	17 (5) ^b^	16 (7)	6 (2)	20 (7) ^b^	18 (8)

^a ^Significant difference (p ≤ 0.05) between groups at baseline

^b ^Significant difference (p ≤ 0.05) between groups at 6 months

^c ^Significant difference (p ≤ 0.05) between groups at 1 year

**Table 4:** Changes in micronutrient (vitamin) intake in both groups from baseline

***Variable per day***	**TFG (n = 35)**	**TFG (n = 35)**	**TFG (n = 35)**	**MRG (n = 35)**	**MRG (n = 35)**	**MRG (n = 35)**
	**Baseline**	**6 month**	**Year 1**	**Baseline**	**6 month**	**Year 1**

Vitamin A (RAE) mcg	604 (314)	795 (401) ^b^	904 (517) ^c^	676 (302)	1480 (671)^b^	1314 (776) ^c^
Beta Carotene (μg)	1693 (1534)	2913 (2236)	3424 (2979)	2455 (2072)	4440 (4429)	3315 (3935)
Vitamin D (mcg)	4 (3)	4 (2) ^b^	5 (9)	5 (2)	8 (3)^b^	7 (3)
Vitamin E (AT) mg	9 (6)	6 (3) ^b^	7 (4) ^c^	10 (5)	11 (8) ^b^	12 (9)^c^
Vitamin K (mcg)	76 (44)	88 (59)	95 (73)	100 (96)	110 (55)	118 (95)
Vitamin C (mg)	65 (38)	103 (60) ^b^	81 (44) ^c^	82 (53)	155 (70)^b^	134 (101)^c^
Thiamin (mg)	1.6 (0.5)	1.2 (0.3) ^b^	1.3 (0.3)	1.6 (0.5)	1.5 (0.4)^b^	1.4 (0.4 0)
Riboflavin (mg)	1.6 (0.5) ^a^	1.5 (0.4) ^b^	1.5 (0.5) ^c^	1.9 (0.6)^a^	1.9 (0.5)^b^	1.8 (0.4)^c^
Niacin (mg)	22 (8)	20 (5) ^b^	20 (6)	22 (7)	24 (7)^b^	22 (7)
Pantothenic Acid (mg)	4 (2) ^a^	4 (1) ^b^	4 (2) ^c^	5 (2) ^a^	8 (2)^b^	7 (2)^c^
Vitamin B6 (mg)	1.6 (0.6)	1.6 (0.4) ^b^	1.6 (0.5) ^c^	1.8 (0.6)	2.3 (0.7)^b^	2.2 (0.7)^c^
Folate (mcg)	340 (138)	315 (90) ^b^	338 (111)	386 (131)	383 (88)^b^	362 (129)
Vitamin B12 (mcg)	3.3 (1.5) ^a^	3.6 (1.7) ^b^	4.0 (3.1)	4.6 (3.0)^a^	5.9 (4.8)^b^	6.3 (8.0)

^a ^Significant difference (p ≤ 0.05) between groups at baseline

^b ^Significant difference (p ≤ 0.05) between groups at 6 months

^c ^Significant difference (p ≤ 0.05) between groups at 1 year

**Table 5:** Changes in micronutrient (mineral) intake in both groups from baseline

***Variable per day***	**TFG (n = 35)**	**TFG (n = 35)**	**TFG (n = 35)**	**MRG (n = 35)**	**MRG (n = 35)**	**MRG (n = 35)**
	**Baseline**	**6 month**	**Year 1**	**Baseline**	**6 month**	**Year 1**

Calcium (mg)	744 (262) ^a^	655 (223) ^b^	659 (207) ^c^	914 (322)^a^	1041 (306)^b^	948 (320)^c^
Phosphorus (mg)	1106 (312)	1026 (241) ^b^	993 (256) ^c^	1260 (334)	1289 (340)^b^	1205 (370)^c^
Magnesium (mg)	2629 (94)	249 (64) ^b^	243 (78) ^c^	289 (90)	393 (109)^b^	374 (133)^c^
Iron (mg)	14 (6)	13 (5)	13 (7)	15 (5)	12 (4)	12 (5)
Zinc (mg)	9 (3) ^a^	9 (3)	9 (3.0)	11 (4)^a^	9 (2)	9 (5)
Copper(mg)	1.2 (0.5)	1.0 (0.3)	1.0 (0.3)	1.3 (0.5)	1.0 (0.3)	1.3 (0.5)
Selenium (mcg)	103 (35)	89 (24)	85 (23)	107 (35)	91 (25)	82 (29)
Sodium (mg)	3054 (993)	2471 (744)	2357 (727)	3468 (1051)	2204 (783)	2230 (838)
Potassium (mg)	2255 (768)	2320 (576)	2232 (728)	2549 (757)	2588 (765)	2472 (987)

^a ^Significant difference (p ≤ 0.05) between groups at baseline

^b ^Significant difference (p ≤ 0.05) between groups at 6 months

^c ^Significant difference (p ≤ 0.05) between groups at 1 year

For the micronutrient mean comparisons between groups, at 6 months, a significant difference (MRG intake greater than TFG) was found for 14 of the essential nutrients, including vitamins (vitamin A, vitamin D, vitamin E, vitamin C, thiamin, riboflavin, niacin, pantothenic acid, vitamin B6, folate, vitamin B12) and minerals (calcium, phosphorus and magnesium). At 12 months there continued to be a significant difference between the groups (MRG mean intake remaining greater than TFG) for 9 nutrients, including vitamin A, vitamin E, vitamin C, riboflavin, pantothenic acid, vitamin B6, calcium, phosphorus and magnesium. Sodium intake was close to the recommendation of < 2300 mg/day in both groups at both time points.

### Nutrient prevalence of inadequacy from three-day food records

Because the US Institute of Medicine (IOM) has recommended that nutrient comparisons may be more appropriately done for groups by comparing the prevalence of inadequacy using the Estimated Average Requirements (EAR) than by comparing mean intakes, this approach was also used in this study [12]. Table 6 defines each of the current DRI measures. In this statistical transformation method, in order to account for the variation in daily intake (comparing the three days of the three-day food records), the proportion below the EAR is counted to determine the prevalence of inadequacy. Therefore, the higher the proportion, the higher the prevalence of inadequacy. We analyzed our data to establish this EAR-cutoff using the recommended SIDE software program from Iowa State University [10]. Our analysis for nutrients with an established EAR or AI (Adequate Intake) are shown in Table 7 and 8. The prevalence of inadequacy of nutrient intake from food as indicated by a level >0.50 at 12 months in the TFG group included calcium (1.00), vitamin E (0.88), pantothenic acid (0.84), folic acid (0.55) and magnesium (0.76). For the MRG, at 12 months the prevalence of inadequacy was calcium (0.70) and vitamin K (0.57), both lower than the prevalence of inadequacy for the TFG group.

**Table 6:** Dietary Reference Intakes (DRI) Definitions

**Estimated Average Requirement (EAR)** The average daily nutrient intake level that is estimated to meet the requirements of half of the healthy individuals in a particular stage and gender group.
**Recommended Dietary Allowance (RDA)** The average daily dietary nutrient intake level that is sufficient to meet the nutrient requirements of nearly all (97–98 percent) healthy individuals in a particular life stage and gender group.
**Adequate Intake (AI)** The recommended average daily intake level based on observed or experimentally determined approximations or estimates of nutrient intake by a group (or groups) of apparently healthy people that are assumed to be adequate, used when an RDA cannot be determined.
**Tolerable Upper Intake Level (UL)** The highest average daily nutrient intake level that is likely to pose no risk of adverse health effects to almost all individuals in the general population. As intake increases above the UL, the potential risk of adverse effects may increase.
**Acceptable Macronutrient Distribution Ranges (AMDR)** The range of intakes of an energy source that is associated with a reduced risk of chronic disease, yet can provide adequate amounts of essential nutrients. The AMDR is expressed as a percentage of total energy intake.
**Estimated Energy Requirements (EER)** The average dietary energy intake that is predicted to maintain energy balance in a healthy individual of a defined age, gender, height, weight, and level of physical activity consistent with good health.

References #11, #12

**Table 7:** Vitamin intake: Estimate of the prevalence of inadequacy at 6 and 12 months

***Variable per day***	**EAR cutoff**	**6 mo TFG**	**6 mo MRG**	**12 mo TFG**	**12 mo MRG**
Vitamin A (RAE) (mg)	500	.39	.01	.41	.12
Vitamin D (mcg)	5*	**.52**	.02	.22	.07
Vitamin E (AT) (mg)	12	**.90**	.10	**.88**	.20
Vitamin K (mcg)	90*	**.67**	**.54**	.46	**.57**
Vitamin C (mg)	60	.01	.05	.30	.22
Thiamin (mg)	0.9	.05	.05	.03	.10
Riboflavin (mg)	0.9	.01	.02	.02	.00
Niacin (mg)	11	.00	.07	.00	.00
Pantothenic acid (mg)	5*	**.94**	.17	**.84**	.14
Vitamin B6 (mg)	1.1	.00	.07	.00	.03
Folate (ug)	320	.46	.20	**.55**	.42
Vitamin B12 (ug)	2	N/A	.02	N/A	.00

EAR = Estimated average requirement

*AI = Adequate intake. No EAR has been established.

RAE = Retinal activity equivalent.

N/A = The adjusted distribution was not available using the cut-point method of analysis.

TFG = Traditional Food Group, MRG = Meal Replacement Group

**Table 8:** Mineral Intake: Estimate of the prevalence of inadequacy at 6 and 12 months

***Variable per day***	**EAR cutoff**	**6 mo TFG**	**6 mo MRG**	**12 mo TFG**	**12 mo MRG**
Calcium (mg)	1000*	**.99**	**.52**	**1.00**	**.70**
Phosphorus (mg)	580	0	.02	.01	.01
Magnesium (mg)	265	**.54**	.12	**.76**	.19
Iron (mg)	8.1	.05	.15	.10	.06
Zinc (mg)	6.8	.11	.19	N/A	.19
Copper (mg)	0.7	.02	.04	.13	.07
Selenium (mcg)	45	.00	.02	.00	.00
Potassium (mg)*	4700*	N/A	N/A	N/A	N/A

EAR = Estimated average requirement

*AI = Adequate intake. No EAR has been established.

N/A = The adjusted distribution was not available using the cut-point method of analysis.

TFG = Traditional Food Group, MRG = Meal Replacement Group

### Fruit and vegetable serving changes

Servings of fruits and vegetables per day are shown in Table 9. At 12 months both groups increased their mean daily intake of fruits and vegetables, from a lower baseline average of 2.9 ± 1.9 to 4.0 ± 2.2 servings/day (+38%) for the TFG, and from a lower baseline average of 3.4 ± 1.8 to 4.6 ± 3.2 servings/day (+35%) for the MRG. At 6 and 12 months, both groups were closer to the recommended 5 total servings of fruits and vegetables per day than at baseline [13].

**Table 9:** Group intakes of fruits and vegetables at baseline, 6 months and 12 months

	**TFG**	**TFG**	**TFG**	**MRG**	**MRG**	**MRG**
***Servings***	**(n = 35)**	**(n = 35)**	**(n = 35)**	**(n = 35)**	**(n = 35)**	**(n = 35)**

***Per day***	**Baseline**	**6 mo**	**12 mo**	**Baseline**	**6 mo**	**12 mo**

**Fruits**	0.8 (.9)	1.7 (1.0)	1.6 (1.4)	1.3 (1.2)	1.9 (1.5)	1.9 (1.9)
*Range*	0.0 – 3.7	0.0 – 4.3	0.0 – 5.7	0.0 – 467	0.0 – 7.6	0.0 – 6.9
**Vegetables**	2.1 (1.4)	2.6 (1.5)	2.5 (1.6)	2.2 (1.3)	2.7 (1.6)	2.7 (1.8)
*Range*	0.3 – 7.0	0.2 – 5.1	0.3 – 8.4	0.0 – 4.7	0.3 – 8.2	0.3 – 8.4
**Total**	2.9 (1.9)	4.4 (1.8)	4.0 (2.2)	3.4 (1.8)	4.5 (2.4)	4.6 (3.2)
*Range*	0.3 – 9.5	0.7 – 8.4	0.5 – 9.5	0.2 – 8.5	1.2 – 11.5	0.3 – 13.2

No significant difference (p ≤ 0.05) between groups was found

Mean ± (standard deviation)

TFG = Traditional Food Group, MRG = Meal Replacement Group

### Use of Meal Replacements

Analysis of the number of servings of meal replacements per day for the MRG showed a mean decrease in use between assessments, with reported levels at 6 months of 1.4 ± 0.6 servings/day (range 0.0 to 2.7), and at 12 months of 1.2 ± 0.7 servings/day (range 0 to 2.0). Although this data indicates that some of the women were consuming the recommended two meal replacements per day, the mean intake was lower than this.

## Discussion

The primary objective of this study was to examine the adequacy of the diet choices by subjects on two different diet interventions for weight loss. Both interventions used the traditional food group approach, with one integrating meal replacements. It is important that nutrient adequacy be measured as a component of the safety of weight loss regimens. Both controlled and uncontrolled weight loss studies using meal replacements have published the diet prescription given to subjects and clinical outcomes. However, few have included the nutrient intake profile of the self-selected diets the subjects actually chose to follow while in the study [4,14-21].

Studies for obese and overweight subjects using a traditional food group system, which is considered the gold standard of dietary instruction that was used as the control in this study, have reported that intake of most nutrients can remain at recommended levels when this approach is taken [22]. For studies using meal replacements, there seems to be an anecdotal concern that these products might not foster nutritional adequacy and balanced food choices long-term. Only two studies published to date by other investigators have analyzed the actual nutrient intake using meal replacements for weight loss. One long-term German study by Ditschuneit *et al *analyzed the seven-day food diaries of 73 obese men and women subjects over a 27 month period [23]. These authors primarily reported on changes in macronutrient intake, summarizing that the fat intake decreased significantly (p ≤ 0.05) from 37.6% to 32.9% in the control group and from 36.1% to 26.4% in the meal replacement group. Dietary cholesterol intake also decreased significantly (p ≤ 0.05) in the meal replacement group (from 422 ± 57 to 244 ± 30 mg/day) compared to the control group (378 ± 43 to 154 ± 14 mg/day). Dietary protein intake was unchanged and was not different between their study groups. A more recent shorter-term Australian study by Noakes *et al *reported on the analyses of three-day food records completed at 4 week intervals by obese or overweight men and women subjects with features of metabolic syndrome (raised triglycerides). Sixty-six matched subjects were randomized into either a meal replacement or control group (prescriptive low fat diet plan) for a six month intervention [24].

The two groups received structured supervision from administrative staff during bi-weekly visits, but they were not given professional nutrition support. The control group was also supplied with shopping vouchers with a similar financial value as the meal replacements. For the 42 subjects (64%) who completed this study, there was no significant difference in weight change or energy intake between the meal replacement group and controls, similar to our study results. This led two of the authors to postulate in a subsequent review article that providing both groups with similar information, structure, support and incentives resulted in a similar weight outcome [25].

Of particular relevance was the finding in the Noakes *et al *study that dietary intake of several essential nutrients (calcium, magnesium, zinc and iron) were significantly higher (p < 0.01) in the meal replacement group compared to the control group. These investigators also measured blood biomarkers to assess fruit and vegetable intake and found that the plasma beta carotene levels increased significantly at six months within the meal replacement group (41% at 6 months, p < 0.001). Although the serum folate and plasma beta-carotene were higher in the meal replacement group, the differences between groups were not statistically significant. Both plasma retinol and alpha-tocopherol, which are stored as fat-soluble vitamins, remained unchanged. The more detailed nutrient analyses of the Noakes *et al *study are in agreement with our findings that the nutritional adequacy of the meal replacement program was equal to or superior to the traditional (control) diet. One caution in interpreting these nutrient intake results in the study by Noakes and colleagues and in our study is that the primary measure of diet adequacy was food records which were kept by the subjects. These self-reports of diet intake are known to have limitations, including underreporting and changes in food selections during the reporting period [25,26]. However, these food record limitations applied to both groups that were compared with regard to their chosen dietary patterns and nutrient adequacy during this weight loss study.

## Conclusion

The results of this one-year intervention study in primarily healthy overweight/obese women showed that both the meal replacement group and traditional food group maintained overall adequate macronutrient and micronutrient intake based on current DRI recommendations. Weight loss and body composition changes indicated that the group interventions were clinically sound and beneficial. Although the women in both groups were instructed on balancing their intake in a series of group discussions led by the same Registered Dietitian, those in the traditional group tended to have significantly lower intakes of a number of essential vitamins and minerals compared to the group using meal replacements. The potentially inadequate intake of calcium is of particular concern since weight-loss diets have been associated with increased bone resorption in obese adults [27]. In conclusion, the results of this study point to the importance of using an adequately fortified meal replacement product and/or taking a multivitamin and mineral supplement to ensure nutrient adequacy during reduced energy intake for weight loss. This advice applies not only to those following a weight loss plan on their own, but also to those following a weight loss plan under professional guidance.

## Competing interests

The author(s) declare that they have no competing interests.

## Authors' contributions

JA as principal investigator designed and coordinated the study intervention and analysis, and drafted the manuscript for submission.

HH assisted with the development of the dietary interventions, carried out the dietary interventions, and helped to draft the manuscript.

SC assisted with all data collection in the study, performed the statistical analysis, and helped to draft the manuscript.

VB assisted with the dietary data collection, development of the dietary interventions, and helped draft the manuscript.

JS assisted with the medical aspects of the interventions and assessments, including review of eligibility, and helped to draft the manuscript.

CP assisted with the laboratory aspects of the intervention and assessments, and helped to draft the manuscript.

All authors read and approved the final manuscript.
